# From nature to nanomedicine: bioengineered metallic nanoparticles bridge the gap for medical applications

**DOI:** 10.1186/s11671-024-04021-9

**Published:** 2024-05-09

**Authors:** Jitendra Patel, G. Shiva Kumar, Harekrishna Roy, Balaji Maddiboyina, Stefano Leporatti, Raghvendra A. Bohara

**Affiliations:** 1 Gitam School of Pharmacy, GITAM (Deemed to be University), Hyderabad Campus, Rudraram, Sangareddy, Hyderabad, TS 502329 India; 2 Department of Pharmaceutics, Nirmala College of Pharmacy, Mangalagiri, Guntur, Andhra Pradesh 522503 India; 3 Department of Medical and Scientific Communications, Scientific Writing Services, Freyr Global Regulatory Solutions & Services, Phoenix SEZ, Hitech City, Gachibowli, Hyderabad, 500081 India; 4 CNR Nanotec-Istituto Di Nanotecnologia, C\O Campus EcotekneVia Monteroni, 3100 Lecce, Italy; 5 D.Y. Patil Education Society (Deemed to be University), Kolhapur, MS India; 6 Present Address: University of Galway, Galway, Ireland

**Keywords:** Nanotechnology, Antimicrobials, Green synthesis, Metallic nanoparticles

## Abstract

The escalating global challenge of antimicrobial resistance demands innovative approaches. This review delves into the current status and future prospects of bioengineered metallic nanoparticles derived from natural sources as potent antimicrobial agents. The unique attributes of metallic nanoparticles and the abundance of natural resources have sparked a burgeoning field of research in combating microbial infections. A systematic review of the literature was conducted, encompassing a wide range of studies investigating the synthesis, characterization, and antimicrobial mechanisms of bioengineered metallic nanoparticles. Databases such as PubMed, Scopus, Web of Science, ScienceDirect, Springer, Taylor & Francis online and OpenAthen were extensively searched to compile a comprehensive overview of the topic. The synthesis methods, including green and sustainable approaches, were examined, as were the diverse biological sources used in nanoparticle fabrication. The amalgamation of metallic nanoparticles and natural products has yielded promising antimicrobial agents. Their multifaceted mechanisms, including membrane disruption, oxidative stress induction, and enzyme inhibition, render them effective against various pathogens, including drug-resistant strains. Moreover, the potential for targeted drug delivery systems using these nanoparticles has opened new avenues for personalized medicine. Bioengineered metallic nanoparticles derived from natural sources represent a dynamic frontier in the battle against microbial infections. The current status of research underscores their remarkable antimicrobial efficacy and multifaceted mechanisms of action. Future prospects are bright, with opportunities for scalability and cost-effectiveness through sustainable synthesis methods. However, addressing toxicity, regulatory hurdles, and environmental considerations remains crucial. In conclusion, this review highlights the evolving landscape of bioengineered metallic nanoparticles, offering valuable insights into their current status and their potential to revolutionize antimicrobial therapy in the future.

**Graphical Abstract:**

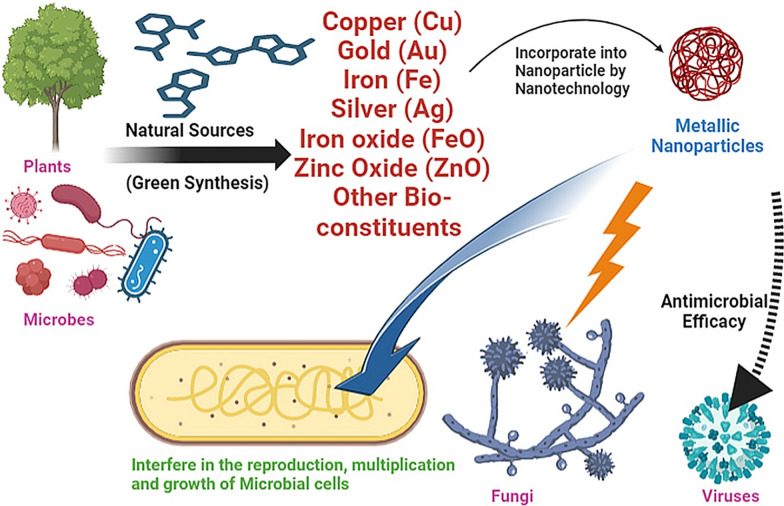

## Introduction

Noble metal nanoparticles, including silver, gold, zinc, selenium, and platinum, hold significant biomedical potential for diagnosing and treating severe conditions such as cancer, HIV, TB, and Parkinson's disease; while silver nanoparticles offer novel antimicrobial properties, gold nanoparticles act as drug carriers, and platinum nanoparticles find applications in bone allografts and dentistry, the discussion also acknowledges toxicity concerns and offers potential solutions to ongoing challenges in the field [1]. In the face of multidrug-resistant organisms posing persistent infectious disease challenges, nanotechnology provides a solution by improving drug absorption, precise delivery, and minimizing side effects; nanoparticles (NPs) can tackle multidrug resistance through multi-component bacterial targeting, synergistically enhancing antibiotic efficacy, while organic NPs offer medical promise due to biodegradability and biocompatibility [2]. Recent findings highlight the potential of combining antibiotics with metallic nanoparticles to sustain bacterial sensitivity to these drugs, offering a promising avenue. Various metallic nanoparticles offer distinct advantages in treating bacterial infections, potentially enhancing the effectiveness of commonly employed antibiotics, even against multidrug-resistant microorganisms [3]. Utilizing green chemistry principles, the synthesis of silver nanoparticles (AgNPs) from diverse biological sources and waste materials is environmentally friendly and cost-effective, resulting in biocompatible nanoparticles with versatile applications as antimicrobial, antifungal, antiviral, and anticancer agents, natural catalysts for pollutant degradation, aid in diabetes-related complications treatment, and facilitation of wound healing [4] as well as in nanocarrier system [5]. The coated cotton fibers exhibited strong antibacterial performance, achieving antimicrobial rates of over 99% even at a minimal Ag content of 3 mg/g. The cooperative self-assembly approach showcased effective AgNP integration into natural fibers, enabling precise control of silver content [6]. The pharmaceutical implications surrounding lipid nanocarriers for transporting and disseminating a range of therapeutic agents, spanning biotechnological products to small pharmaceutical molecules, represent a burgeoning field of interest. These lipid nanoparticles, employed as drug delivery systems, boast a multitude of enticing attributes, including exceptional biocompatibility, ease of synthesis, tissue specificity, evasion of the reticuloendothelial system, controlled drug release, scalability, non-toxicity, and precision-targeted delivery [7]. Biodegradable polymeric nano-based targeted drug delivery technologies have demonstrated remarkable advantages in achieving precise local delivery while minimizing unintended off-target side effects [8]. Solid lipid nanoparticles (SLNs) show promise in drug delivery due to enhanced stability and controlled release. Yet, achieving precise organ and site-specific delivery is a complex task. Overcoming barriers like particle size optimization and evading defense mechanisms is essential. Tailoring SLNs to distinct organs is ongoing research, holding great potential [9].

## Antimicrobial nanoparticles from Green synthesis

**Fig. 1:**
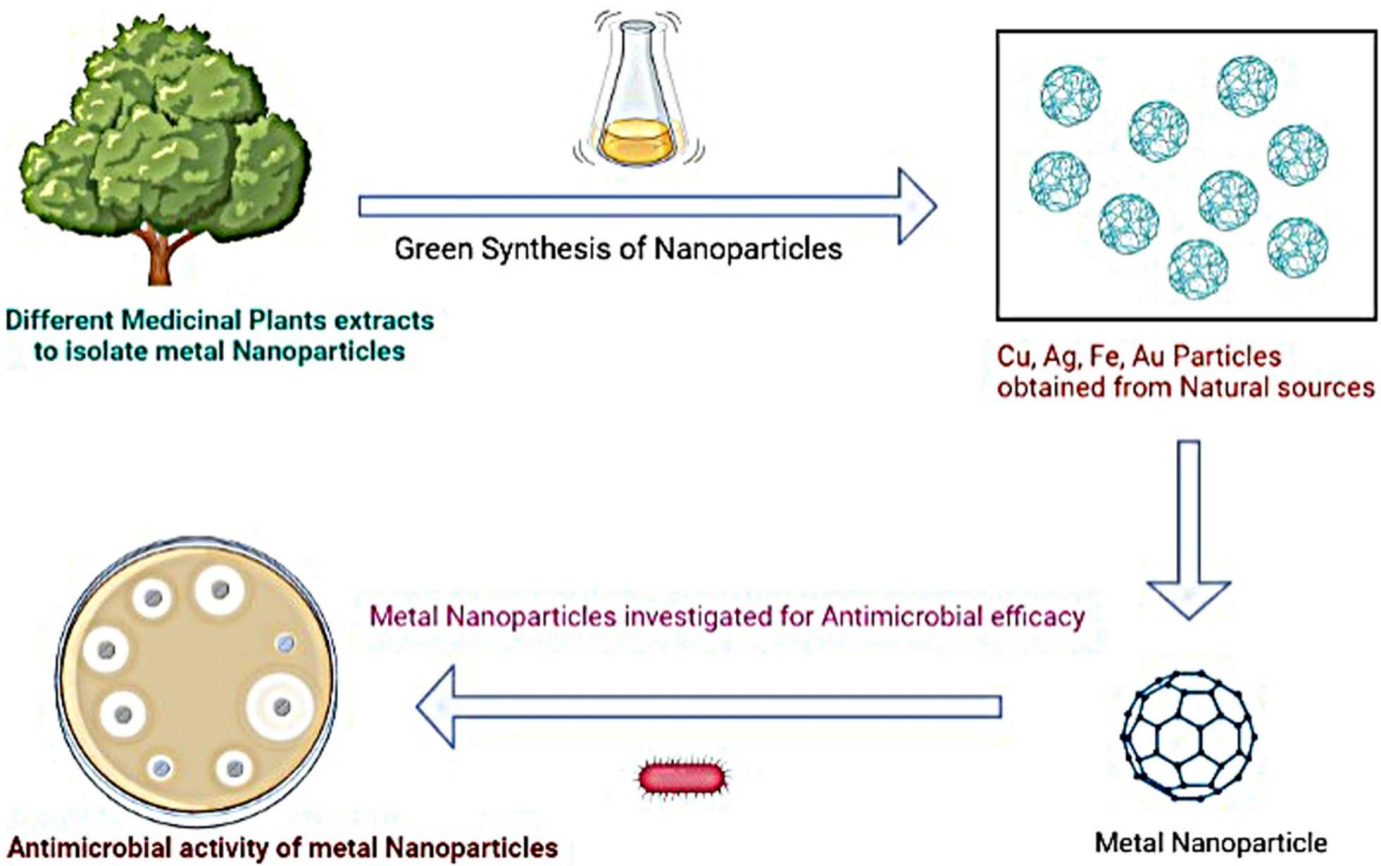
Antimicrobial nanoparticles derived from environmentally friendly or 'green' synthesis methods

Utilizing the principles of green synthesis, the fabrication of nanoparticles and their subsequent application in the realm of applied health is a field of growing significance. This encompasses a spectrum of areas including biosensors, drug delivery systems, cancer therapy modalities, and antimicrobial interventions [10]. *Garcinia cambogia*, along with Merleberry, Malabar Tamarind, Goraka, and Kudam Puli, is imbued with pivotal metabolites that orchestrate the reduction of metal ions and concomitant stabilization of nanoparticles, constituting the mechanistic underpinning of the synthesis process [11]. Those nanoparticles developed from various plant sources utilizing suitable technique depicted in Fig. 1.

### Copper nanoparticles

The green synthesis approach for producing copper nanoparticles (CuNPs) involves the extraction of *P. austroarabica* (*P. austroarabica*). These CuNPs exhibit notable antioxidant activity and hold promising potential as therapeutic agents for human breast cancer treatment. Furthermore, they display remarkable efficacy in combating bacterial and fungal infections, effectively targeting organisms such as *Streptococcus aureus*, *Escherichia coli*, *Penicillium chrysogenum*, and *Fusarium oxysporum* [12].

### Gold nanoparticles

*Origanum vulgare* leaves and stems harbor essential oils, flavonoids, phenols, and anthocyanins, while the *O. vulgare* extract (OVE) serves as a green synthesis route facilitated by biocompatible gold nanoparticles (AuNPs), boasting antioxidative, antimicrobial, and plasmonic attributes [13].

### Iron nanoparticles

The green synthesis of iron nanoparticles of isolated proanthocyanidins may provide a new opportunity for cancer treatments to minimize the toxic effects of available cancer drugs specifically aimed at a specific location [14].

### Silver nanoparticles

*Cassia auriculata* extracts (CAs) hold promise for silver nanoparticle (AgNPs) synthesis, exhibiting potent antimicrobial and antioxidant potential, with biosynthesized AgNPs demonstrating concentration-dependent effects against *Bacillus cereus, Escherichia coli, Klebsiella pneumoniae*, and *Staphylococcus aureus* bacteria [15]. The eco-friendly production of silver nanoparticles (CTAgNP) using *Cullen tomentosum* (Thunb.) acetone extract, accompanied by evaluating the plant extract and biogenic nanoparticles' antibacterial efficacy against *Bacillus cereus* and *Staphylococcus aureus*, Gram-positive bacterial strains [16]. In order to cure wounds, bacterial cellulose (BC) impregnated with green synthesised silver nanoparticles (AgNPs) is being tested as an antibacterial membrane [17]. Utilizing *Anthemis pseudocotula* Boiss. plant extract, silver nanoparticles (AP-AgNPs) are synthesized through a cost-effective and eco-friendly process, with pronounced antibiotic activity primarily affecting Gram-negative bacteria MDR-PA compared to other microbial strains [18]. Both in vitro and in greenhouse trials, biosynthesized AgNPs were found to be significantly effective against bacterial, fungal, and viral phytopathovars, and they were considered to be capable agrichemical alternatives for bactericides, fungicides, and nematocides [19]. In order to eliminate plant infections, silver's antimicrobial characteristics have been repurposed into nanoparticles (AgNPs) created using physiochemical synthesis [20]. Utilizing natural extracts, metal and metal oxide nanoparticles were produced, including gold (Au), silver (Ag), copper oxide (CuO), and zinc oxide (ZnO) [21]. Instead of using chemical reduction agents, biological processes are used to transform silver ions into silver nanoparticles. The plant extracts that are utilized to make silver nanoparticles with antibacterial properties [22].

The potential of biodegradable trash is developed as a workable substitute to develop a sustainable economy that benefits all people by using it to prepare nanomaterials [23]. *Nymphae odorata* plant extract was used as a reducing and capping agent during the green method's formulation of silver nanoparticles (AgNPs) [24]. Utilizing the renewable *Cassia fistula* extract as a powerful capping and stabilising agent, biofriendly and environmentally friendly methods are developed to create zinc oxide nanomaterial [25]. Due to the rising demand for silver, a noble metal with special qualities and potential medical uses, new and appropriate production methods are needed [26]. The use of various extracts from several Euphorbia plant species to produce silver nanoparticles in a sustainable manner. The properties of biosynthesized silver nanoparticles, including their analgesics, antiviral, anticoagulant, antifungal, antiparasitic, nematicidal, anticancer, anti-inflammatory, antiplasmodial, antioxidant, and larvicidal effects [27–30]. Aqua extract from *Raphanus sativus* L. (RS) leaf was used to create plant-based AgNPs. The human colon cancer cell (CaCo-2) has been discovered to be susceptible to treatment with plant-based AgNPs, which also significantly inhibited the development of *Candida albicans* and *Staphylococcus aureus* [31]. *Grewia asiatica* L. leaf extract, which was discovered to be a highly powerful antibacterial and larvicidal chemical, was used to create copper nanoparticles (CuNPs) [32]. Utilizing *Prunus persica* L. (peach pomace) as a natural deep eutectic solvent, along with a plasma-liquid method, silver nanoparticles (AgNPs) were synthesized, demonstrating significant antibacterial activity against pathogenic *Escherichia coli*, *Staphylococcus aureus*, and *Candida albicans* at moderate concentrations [33].

A variety of worldwide flora-fabricated silver nanoparticle phytosynthesis was created. It has emerged as a green nanoweapon for SARS-CoV-2 and other antiviral strategies [34]. By utilising the reducing and capping potential of *Mussaenda frondosa* leaf, stem, and callus aqueous extracts, zinc oxide nanoparticles (ZnO-NPs) were biosynthesized [35]. Due to their safety and environmental friendliness, biosynthesized silver nanoparticles (Ag NPs) show potential uses in a variety of disciplines. It has been outlined how Ag NPs have been used in various stem, fruit, and seed components, as well as how this has affected the morphological characteristics [36]. Water extracts from the peel and flesh of two *Pyrus communis* L. cultivars, Forelle (Red) Pears (RPE) and *Packham Triumph* (Green) Pears (GPE), were employed for the synthesis of silver nanoparticles (AgNPs), demonstrating their potential utility for treating bacterial infections in biological environments [37]. *Paederia foetida* L. leaf extract in the green synthesis of silver nanoparticles and evaluation of their antimicrobial activities. The silver nanoparticles as they were created demonstrated outstanding antibacterial activity against numerous gramme classes of bacteria and would make a promising candidate for a number of pharmacological, biological, and environmental uses [38]. The production of antimicrobial silver nanoparticles by the strain *W. oryzae* DC6 may be made easy, environmentally friendly, economical, dependable, and safe [39]. Cerium oxide nanoparticles (CeO_2_NPs) produced by green synthesis have antibacterial uses. It offers a logical understanding of the significant achievements of CeO_2_ NPs as innovative therapeutic agents for treating a variety of microbial diseases and other infections [40]. Whether coffee and green tea extracts are appropriate for using in the green synthesis of silver nanoparticles as well as how the produced materials behave in various biological systems. The fact that these particles were extremely hazardous to mammalian cells despite having outstanding antibacterial activity against all of the tested microbial diseases restricts their potential applications [41].

## Antimicrobial formulations by nanotechnology

### Metallic nanoparticles for antimicrobial

**Fig. 2:**
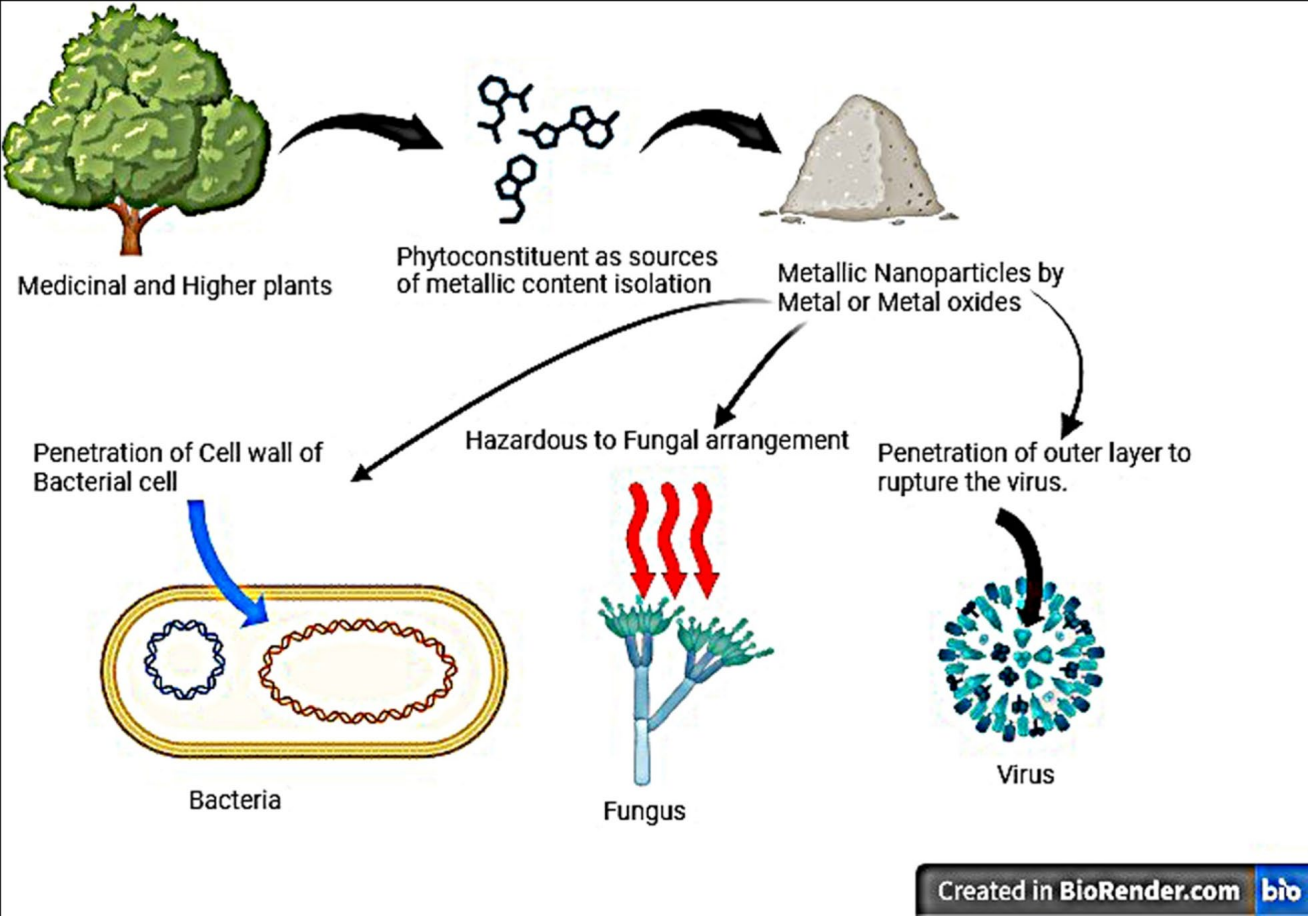
Nanoparticles composed of metals with inherent antimicrobial properties, engineered for combating microorganisms

Utilizing bioactive components from sustainable plant sources, such as essential oils, phenolics, flavonoids, alkaloids, glycosides, and saponins, offers a diverse range of applications, including antibacterial, anticancer, and antioxidant qualities, along with their potential for food preservation and biosynthesis of metallic nanoparticles as seen in Fig. 2, thereby providing ecologically friendly alternatives to conventional pesticides and preservatives [42]. Utilizing metallic nanoparticles and nanosystems, including core–shell nanosystems with attached antimicrobial agents, holds promise for enhancing antimicrobial efficacy against planktonic and biofilm microorganisms, circumventing microbial resistance, minimizing cellular toxicity, and potentially enabling antimicrobial dosage reduction [43]. The past decade has witnessed a significant surge in the application of metals, metal oxides, and their integration into polymers, which exhibit pronounced antibacterial, antifungal, and antiviral effects, thereby holding promise for addressing oral infections; nanoparticles, renowned for their biocidal, anti-adhesive, and transport attributes, offer the potential to regulate oral biofilm growth, rendering this area of research increasingly captivating, with a focus on recent advancements including nanoparticle employment in photodynamic therapy and exploration of methods to enhance biocompatibility and targeted functionality [44].

### Zinc oxide nanoparticles for antimicrobial

**Fig. 3:**
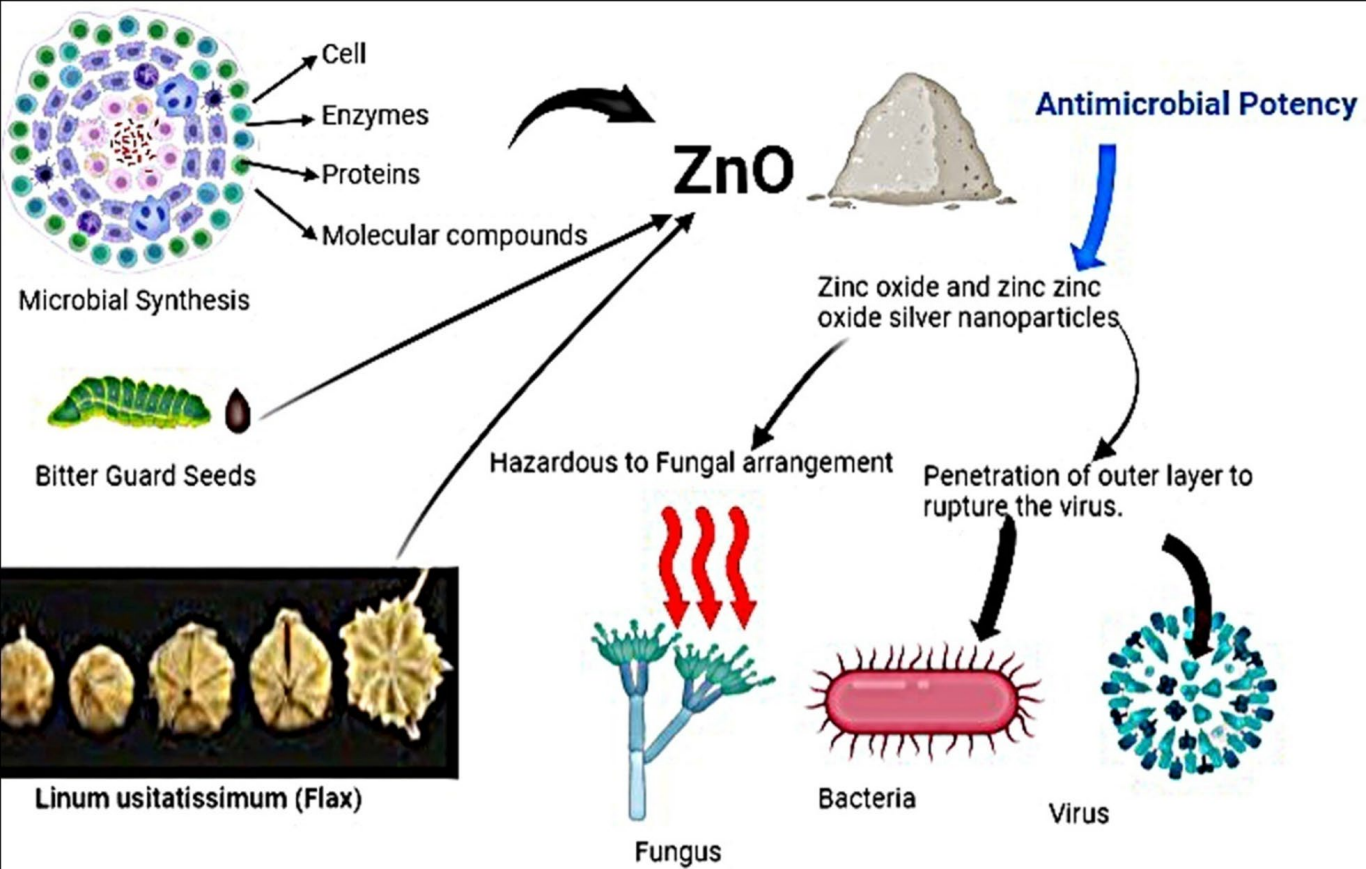
Utilizing zinc oxide nanoparticles with potent antimicrobial attributes for combating microbial pathogens

Utilizing microbial cells, enzymes, proteins, and molecular compounds, zinc oxide nanoparticles are synthesized via intracellular or extracellular pathways by prokaryotic and eukaryotic microorganisms including bacteria, fungi, and yeast, showcasing size-dependent antibacterial properties with distinct applications as narrated in Fig. 3; ZnO NP biosynthesis mechanisms, optimization criteria, and potential roles as antibacterial agents and animal feed supplements, alongside associated toxicological risks in agriculture [45]. Employing zinc oxide (ZnO) and zinc oxide-silver (ZnO-Ag) nanoparticles, two varying concentrations (0.5 and 1 wt%) were incorporated into poly(butylene adipate-co-terephthalate) (PBAT) to form antimicrobial films [46]. Employing the endophyte *Trichoderma viride*, isolated from *Momordica charantia* seeds, ZnO nanoparticles (NPs) were biogenically synthesized, exhibiting a hexagonal morphology with an average particle size of approximately 63.3 nm; notably, these NPs demonstrated substantial antibacterial efficacy against multi-drug resistant organisms upon testing [47]. The antimicrobial potential of *Linum usitatissimum* was assessed in the presence of ZnO/Zn(OH)2 nanoparticles, obtained through maceration to produce hydroalcoholic extracts, and the resulting extracts were examined for microbial growth inhibition using broth macrodilution and agar disc diffusion methods, highlighting the prospect of utilizing plant extracts combined with metal oxide nanoparticles as a robust alternative to antibiotics for combating bacterial infections [48].

*Botryococcus braunii* was employed as a bioreactor to produce antimicrobial nanoparticles, with microalgae generating metabolites that act as eco-friendly reducing and capping agents for silver nanoparticle synthesis; these AgNPs were synthesized using live *Botryococcus braunii* cells and exhibited notable antimicrobial activity, especially against Gram-negative bacterial strains [49]. Utilizing a micro/nanoencapsulation approach emerges as a promising strategy to enhance the efficacy of natural antimicrobials against *Listeria monocytogenes* in food products, addressing the threat of listeriosis from this pathogenic bacterium, known for its adhesion capabilities and resilience to food processing conditions, thereby offering an appealing alternative to synthetic preservatives in organic food production [50]. It is also seen that, secondary metabolite conjugation with the surface of inorganic nanoparticles interferes directly with the biological activity of inorganic nanoparticles and not only may enhance the bioactivities of these inorganic nanoparticles but also may increase or decrease the inorganic nanoparticles side effects. This provides an opportunity for us to select a suitable natural resource for preparation of inorganic nanoparticles [51].

### Plant-based nanoparticles for antimicrobial activity

Flavonoids, amino acids, proteins, polysaccharides, enzymes, polyphenols, and reducing sugars present in plant extracts facilitate the simplification of nanoparticle reduction, synthesis, and stabilization processes, leading to the creation of nanoparticles endowed with antioxidant, antibacterial, antifungal, and anticancer properties [52]. The integration of Curcumin-Nisin-poly (L-lactic acid) nanoparticles (CurNisNps) into orthodontic acrylic resin was investigated to assess its mechanical attributes and antimicrobial impact against *Streptococcus mutans* and *Candida albicans*, revealing a potent enhancement to the acrylic resin's antibacterial efficacy without compromising its mechanical integrity [53]. Exploring lignin's multifunctional roles as a UV-blocking, antioxidant, and antimicrobial agent, this review highlights the latest advancements in the creation and advanced applications of packaging films derived from lignin, encompassing its utilization from both non-wood and wood sources to develop biodegradable film solutions tailored for food packaging purposes [54].

Utilizing CuS nanoparticles and *Linum usitatissimum* root and shoot extracts, the antimicrobial potential was evaluated through hydroalcoholic extracts prepared via maceration, wherein the combined effects were tested against various pathogens using broth macrodilution and agar disc diffusion, including determination of minimal inhibitory and bactericidal concentrations [48]. Iron oxide nanoparticles loaded with Himalayan honey, a natural derivative from the wild honey bee population in Nepal's Himalayan highlands with historical medicinal applications, could open up novel opportunities for developing hybrid nanomaterials in the field of biomedicine, leveraging the synergy between nanotechnology advancements and the beneficial attributes of honey [55]. Utilizing plant-mediated methods, the synthesis of zinc oxide nanoparticles has garnered significant interest due to their multifunctional properties, notably their antimicrobial efficacy, presenting a reliable, cost-effective, biocompatible, and environmentally friendly approach through green synthesis techniques [56].

The "encapsulate-and-deliver" strategy utilizing inorganic hollow mesoporous spheres offers a means to achieve controlled release, preventing resistance due to excessive dosing, making them a promising and feasible choice for effective antimicrobial delivery, considering factors like loading capacity, engineering feasibility, and cost-effectiveness [57]. Harnessing the potent antibacterial attributes and limited resistance potential of antimicrobial peptides (AMPs), nanomedicine-based AMP delivery methods, encompassing metallic, lipid, and polymeric nanoparticles, along with hybrid systems, hold promise for enhancing antimicrobial efficacy and offer potential alternatives to antibiotics against bacterial infections [58].

**Fig. 4:**
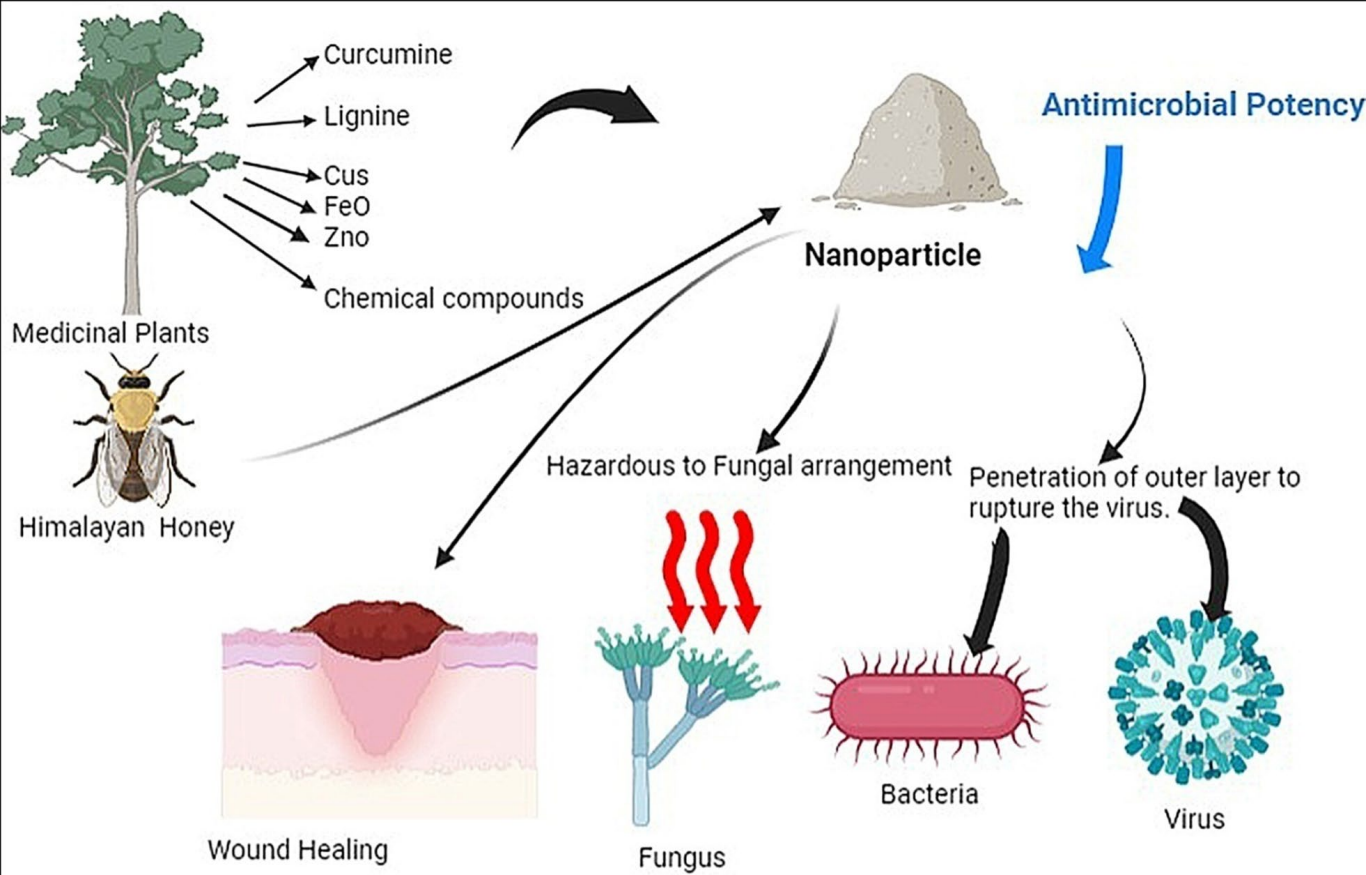
Exploiting nanoparticles derived from plants to harness their natural antimicrobial properties and enhance their effectiveness

Nanomaterials, ranging in size from 1–100 nm, have emerged as unique antibacterial agents, offering several classes of antimicrobial nanoparticles (NPs) and nanocarriers for antibiotic delivery that exhibit efficacy against infectious diseases as seen in Fig. 4, including antibiotic-resistant strains, demonstrated through in vitro and animal model studies, thus presenting potential advantages over conventional drugs due to their distinctive properties stemming from high surface area-to-volume ratios [59]. Efficient neutralization of nitro-based antimicrobial agents, furazolidone and chloramphenicol, was achieved by utilizing rhenium oxide nanoparticles stabilized with monosaccharide and polysaccharide biopolymers, with the preparation of these involving a two-step process tailored for this application [60].

Antimicrobial cryogel dressings have emerged as a viable solution to enhance wound healing by minimizing infections and promoting expedited recovery [61]. While topical antimicrobial agents are effective in managing wound infections, it is essential to consider their selection wisely among options like biguanide, silver, iodine, chlorine compounds, antiseptics, organic agents like honey and curcumin, and antibiotics such as bacitracin, mafenide, mupirocin, neomycin, and silver sulfadiazine, with careful attention to avoiding inappropriate antibiotic use and ensuring proper choice for specific populations [62].

Enhancing the antimicrobial efficacy of nanoparticles is achieved through modulation of their optical and dielectric characteristics, where chemical doping and coating techniques are employed to tailor the physical, chemical, and biological attributes for specific applications, demonstrated here via cost-effective solvothermal synthesis producing nickel-doped cupric oxide nanoparticles with cetyltrimethylammonium bromide stabilizer caps, allowing observation of their structural, morphological, optical, dielectric, and antibacterial properties [63]. In response to the demand for minimally processed, safe, and high-quality foods, the use of natural antimicrobial agents has garnered significant attention in food science, with nanostructures providing a compelling avenue for controlled release, protection, and distribution of substances like bacteriocins and antimicrobial proteins, offering a means to effectively combat pathogenic and spoilage microorganisms while ensuring stability in food handling and storage [64]. While developing nanoparticles, it also takes into consideration which can enhance the therapeutic benefit including morphology, surface area, surface energy and charges [65]. While developing it is also necessary to be tested in biological model which can justify biocompatibility, sustainability, stability issue, and also provide information on unnecessary processing during synthesis.

Electrospun nanofibers (NFs) offer a versatile platform for antimicrobial delivery, showcasing diverse NFs with inherent antibacterial properties that have been introduced and evaluated, hinting at the imminent potential for their clinical application and commercial production [66]. Leveraging the encapsulation technique of nanoparticles emerges as a strategy to preserve bioactive chemical antibacterial attributes and enable controlled release, as explored in this article that delves into the potential of combining natural antimicrobials with nanotechnology to effectively sanitize food surfaces and impede the formation of microbial biofilms [67].

Addressing foodborne pathogens, which pose significant threats to food safety and human health, nanomaterials exhibit potent bactericidal properties and stability, offering potential solutions for controlling these challenging microorganisms in the food industry, as revealed in recent studies that shed light on enhancing the efficacy of natural antibacterial agents through synergistic interactions with nanoparticles [68]. Nanotechnology's appeal lies in crafting materials with enhanced qualities, while the antimicrobial potential of nanoparticles is underscored, considering the processes of bacterial resistance, the modes of action of nanometals and oxide nanoparticles as antimicrobials, and the implications of creating nanoparticle-resistant microorganisms, along with associated human health concerns [69]. Current strategies to investigate the antimicrobial potentials of nanoparticles involve exploring their effectiveness against both viral and bacterial infections, with nanoparticles (NPs) demonstrating unique attributes that enable them to induce cellular damage through diverse mechanisms, resulting in potent antibacterial effects across a range of diseases [70].

In light of the limited and increasingly ineffective antifungal medications, the escalating threat of fungal infections to human health and well-being prompts exploration of inorganic nanoparticles as alternative treatments, as discussed in recent research highlighting their potential efficacy in combating fungal diseases and addressing drug resistance concerns [71]. The amalgamation of antimicrobial photodynamic therapy and nanotechnology presents a potent strategy for infection control, circumventing microbial resistance; leveraging the nanoparticles' attributes crucially impacts selectivity and efficacy, demanding a comprehensive grasp of the target microorganism and its surroundings for optimal nanocarrier selection [72].

### Silver and gold nanoparticles for antimicrobial.

Utilizing phytosynthesis, a rapid, cost-effective, and scalable method, silver and gold nanoparticles were extracellularly synthesized using aqueous leaf extracts from the *Haloxylon salicornicum* plant, demonstrating significant biocidal efficacy against Gram-positive bacteria such as Staphylococcus aureus and Bacillus cereus in contrast to Gram-negative bacteria like Escherichia coli and *Klebsiella pneumoniae* when exposed to 1 mg/mL concentrations [73]. By integrating silver and gold nanoparticles into advanced hydrogels, researchers address both the potent antibacterial and antibiofilm properties of the nanoparticles and the efficient transport facilitated by hydrogels, thus providing a promising strategy to combat infections caused by multidrug-resistant pathogenic microorganisms through a porous hydrogel structure with high water retention capacity [74]. Utilizing a novel lipopeptide biosurfactant derived from *Pseudomonas* sp. OXDC12, silver nanoparticles were synthesized, demonstrating robust colloidal stability and exhibiting highly potent antimicrobial and antifungal activity against *Candida albicans, Fusarium oxysporum, Staphylococcus aureus, Salmonella typhimurium, Klebsiella pneumoniae*, and *Escherichia coli* [75].

Biological fabrication of metallic nanoparticles, encompassing a spectrum including gold (Au), silver (Ag), copper oxide (CuO), zinc oxide (ZnO), iron (Fe2O3), palladium (Pd), platinum (Pt), nickel oxide (NiO), and magnesium oxide (MgO), has been pursued through empirical investigations involving flora, fauna, and microorganisms spanning a wide taxonomic hierarchy [76]. Given the economic impact of *Streptococcus agalactiae*-induced cow mastitis, the escalating antimicrobial resistance in dairy farms has prompted heightened interest in alternative therapies such as natural herbal antimicrobials and nanoparticles; this study investigates the antibacterial and antibiofilm efficacy of cinnamon oil, silver nanoparticles (AgNPs), and their combined application against multidrug-resistant *S. agalactiae* isolated from clinical bovine mastitis in Egypt [77]. Addressing the antibiotic-resistant Gram-negative pathogen *Burkholderia pseudomallei*, which causes melioidosis, this study introduces andrographolide-stabilized silver nanoparticles (andro-AgNPs, with an average diameter of 16 nm) that display remarkable antimicrobial efficacy against *B. pseudomallei* strains, including ceftazidime-resistant variants, surpassing the effectiveness of ceftazidime and other environmentally synthesized AgNPs by 1–3 and 1–2 orders of magnitude, respectively [78].

**Fig. 5:**
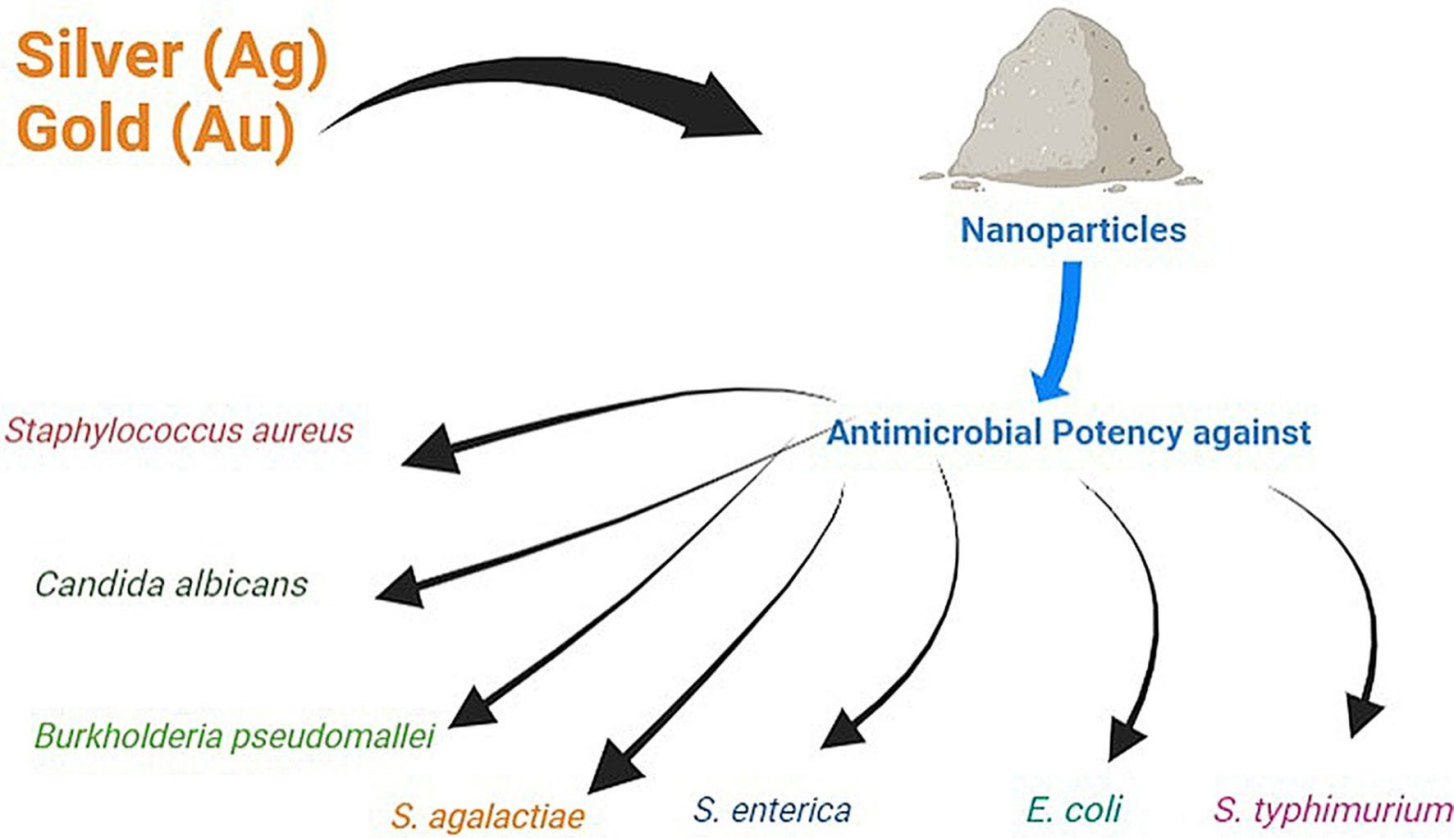
Employing silver and gold nanoparticles renowned for their antimicrobial properties, to combat and inhibit the growth of microorganisms

Enhancing cotton fabric's antimicrobial properties, this study employed silver nanoparticle impregnation via chitosan (Cs) or chitosan-organosilica (Cs-OSH) solutions as adhesive matrices; the antibacterial efficacy of resulting fabrics was evaluated using the agar diffusion method, with a recommendation to opt for the chitosan-organosilicon matrix for improved and sustained bonding between AgNPs and cotton fibers, thereby enhancing antibacterial activity [79]. Silver nanoparticles (AgNPs) were synthesized using *Talinum triangulare* leaf extract, followed by characterization; their antimicrobial effectiveness was evaluated against Gram-positive bacteria (*Bacillus subtilis* and *Staphylococcus aureus*), Gram-negative bacteria (*Escherichia coli*, *Salmonella typhi*), and Gram-neutral fungus (*Candida albicans*), alongside assessing the leaf extract and AgNO3 for comparison [80]. Utilizing the endophytic fungus *Crypto sporiopsisericae* PS4, derived from the medicinal plant *Potentilla fulgens* L., silver nanoparticles (AgNPs) were biosynthesized, averaging 5.5 3.1 nm in size; these AgNPs were tested individually and in combination with chloramphenicol/fluconazole against *Staphylococcus aureus*, *Salmonella enterica*, *Escherichia coli*, *Enterococcus faecalis*, and *Candida albicans*, revealing potential antibacterial efficacy as explained in Fig. 5 [81].

In response to the escalating need for antibacterial characteristics in cellulose-based textiles, the integration of silver nanoclusters/silica composite coating onto cotton fabric has been achieved using the RF co-sputtering technique, presenting an effective approach. The broad exploration of silver's antibacterial potential offers versatile applications through both wet and dry methodologies [82].

Harnessing gelatin's remarkable biocompatibility and biodegradability, which positions it as a highly promising biomaterial, the synthesis of antimicrobial gelatin nanofibers incorporated with silver nanoparticles was achieved via electrospinning a formulation comprising gelatin, AgNO_3_, and formic acid, further bolstered by UV radiation to enhance the antibacterial efficacy of the gelatin structure [83]. *Prunella vulgaris*, recognized as self-heal and valued in medicine for its chemical constituents like Prunellin from the Lamiaceae family, underwent experimentation on calli cultures with different gold (Au) and silver (Ag) nanoparticle ratios to assess antibacterial attributes, unveiling its pivotal role in developing nanotechnology-driven antimicrobial treatments with versatile applications in pharmaceutical research [84]. Utilizing *Morus alba* leaf extract from the S_1_ genotype, this study showcases the biogenic synthesis of silver nanoparticles and assesses their antimicrobial and antioxidant efficacy, wherein optical analysis revealed an SPR peak within the 423–450 nm range confirming nanosilver synthesis; furthermore, these nanoparticles displayed notable metal chelation and dose-dependent antioxidant activity against various free radicals including DPPH, ABTS + , superoxide, and nitric oxide [85].

While nanotechnology's applications have traditionally centered on medicine and pharmaceuticals, its recent extension into agriculture and food industries has prompted an investigation into the impact of silver nanoparticles on probiotic bacteria, utilizing *Lactobacillus acidophilus* LA-5, *Bifidobacterium animalis* subsp. lactis BB-12, and *Streptococcus thermophilus* ST-Y31, all derived from fermented milk products [86]. Leveraging the antimicrobial properties of *Martynia annua*, silver nanoparticles (Ag NPs) were biosynthesized using its aqueous plant extract, with high-resolution transmission electron microscopy (HRTEM) revealing spherical Ag NPs ranging from 10 to 25 nm; both the extract and green-synthesized Ag NPs demonstrated potent antibacterial and antifungal activity against various microorganisms [87]. Leveraging the distinctive phytochemistry of *Dioscorea bulbifera*, silver nanoparticles were synthesized by reducing Ag( +) ions with an extract from its tubers; while the antibacterial impact of the silver nanoparticles showed resistance against both Gram-negative and Gram-positive bacteria, their combination with macrolide (erythromycin) and beta-lactam (piperacillin) antibiotics displayed notable enhancements of three and six times, respectively [88]. Mono and bimetallic silver/gold nanoparticles emerge as antimicrobial agents, capitalizing on the biocompatibility of gold nanoparticles (AuNPs) and the reduced toxicity of silver nanoparticles (AgNPs) towards healthy human cells, resulting in predicted superior therapeutic efficacy; a novel, efficient, and environmentally friendly process facilitated the creation, characterization, and assessment of these nanoparticles for diverse anti-infection biomedical applications [89]. The potential therapeutic application of plant-mediated silver nanoparticles lies in their environmentally benign green synthesis process, ensuring safety, non-toxicity, and enhanced biocompatibility, rendering them suitable for medical use [90]. There have been several biocompatibility studies conducted which reported acceptable sensitization study, irritation study, toxicity, genotoxicity and hemocompatibility with biological cells [91]. The antimicrobial potential of silver nanoparticles (AgNPs), synthesized by reducing Ag + ions in a *Bacillus aerius* culture's cell-free supernatant and using silver nitrate (AgNO3) as a precursor, was exhibited as potent antibacterial activity against multidrug-resistant bacterial pathogens across a spectrum of infections [92].

## Biobased nanotechnology for addressing antimicrobials

### Plant mediated formulations

The environmentally friendly approach of plant-mediated green synthesis produces silver nanoparticles (AgNPs) with sizes ranging from 1 to 100 nm, exhibiting potential applications including antimicrobial, antioxidant, and anticancer activities, highlighting their distinct roles across diverse fields [93]. Exploring nanobiotechnology's submicroscopic structures (1-100 nm), the versatile zinc oxide (ZnO) nanoparticles synthesized using an eco-friendly approach involving zinc-acetate-dihydrate and *Lallemantia royleana* seed extract exhibit antimicrobial properties, catalytic potential, and UV-blocking abilities, offering potential for industrial pollutant degradation and purification applications [94]. Amid the increasing demand for rapid nanotechnology progress, silver nanoparticles play a pivotal role in sectors such as medicine, textiles, and home appliances, given their unique physicochemical properties and antibacterial characteristics; the environmentally conscious green synthesis method employs *Aloe vera* and *Thuja orientalis* leaf extracts, harnessing their diverse medical compounds for nanoparticle production [95].

Utilizing natural deep eutectic solvents (NDES) such as citric acid-propanediol-proline, antimicrobial selenium nanoparticles are synthesized from *propolis* extract and quercetin, yielding enhanced content of active compounds compared to water extraction, particularly beneficial for poorly water-soluble components, with resulting nanoparticles exhibiting confirmed antimicrobial activity against *E. coli, P. aeruginosa, S. aureus,* and *C. albicans*, while the *propolis* extract achieved its highest content of flavonoids, terpenes, and antioxidants through this method [69]. Chia seed's aqueous extract acts as both a reducing agent and stabilizer for biologically synthesized silver nanoparticles (AgNPs) at room temperature, displaying potent antibacterial efficacy even at low concentrations (10 μg), while providing an environmentally friendly approach that enhances the AgNPs' antioxidant, reduction potential, catalytic, and antibacterial attributes [96]. Ultra-small silver nanoparticles (AgNPs) with sizes averaging between 14 to 20 nm were efficiently synthesized from pineapple peel waste (*Ananas comosus*) aqueous extract, obviating the requirement for supplementary reducing or stabilizing agents; these AgNPs effectively eliminated pathogenic bacteria *Pseudomonas aeruginosa* and *Bacillus subtilis*, verified through zone of inhibition (ZoI) and viability assessments [97]. Silver nanoparticles with potential for biosynthesis were created using the aqueous seed extract of *Nigella sativa* (Bc-AgNPs). These nanoparticles displayed noteworthy antibacterial effects against Gram-negative bacteria and exhibited 89% scavenging activity against free radicals, particularly at lower concentrations [98].

Leveraging the fungus *Shizophyllum commune*, silver nanoparticles (AgNPs) and copper nanoparticles (CuNPs) have been biologically synthesized, potentially offering antimicrobial efficacy against multidrug-resistant (MDR) microorganisms. These nanoparticles hold promise for diverse biomedical applications against pathogenic microbes and the management of fungal strains, showcasing their innovative potential in addressing health-related challenges [99]. Orange peel aqueous extract (OPE) is employed for eco-friendly synthesis of silver nanoparticles (AgNPs) with potent activity against diverse pathogens and rat glial tumor cells, offering an affordable green technology method applicable to biomedical, cosmetic, and pharmaceutical sectors [100]. Utilizing aqueous extract from *Phlogacanthus turgidus* leaves, a successful synthesis of silver nanoparticles (AgNPs) and gold nanoparticles (AuNPs) was achieved, wherein PT-AgNPs exhibited potent antibacterial activity against *Bacillus subtilis, Staphylococcus aureus, Salmonella typhi,* and *Escherichia coli* strains [101]. Utilizing *Bacillus thuringiensis* kurstaki (Btk), silver nanoparticles (Btk-AgNPs) were eco-friendly and economically synthesized as a nanobiopesticide, demonstrating superior virulence towards cabbage looper larvae (*Trichoplusiani*) over black cutworm larvae (*Agrotisipsilon*), with notable efficacy using Bt supernatant or pellet for AgNP production [102].

**Fig. 6:**
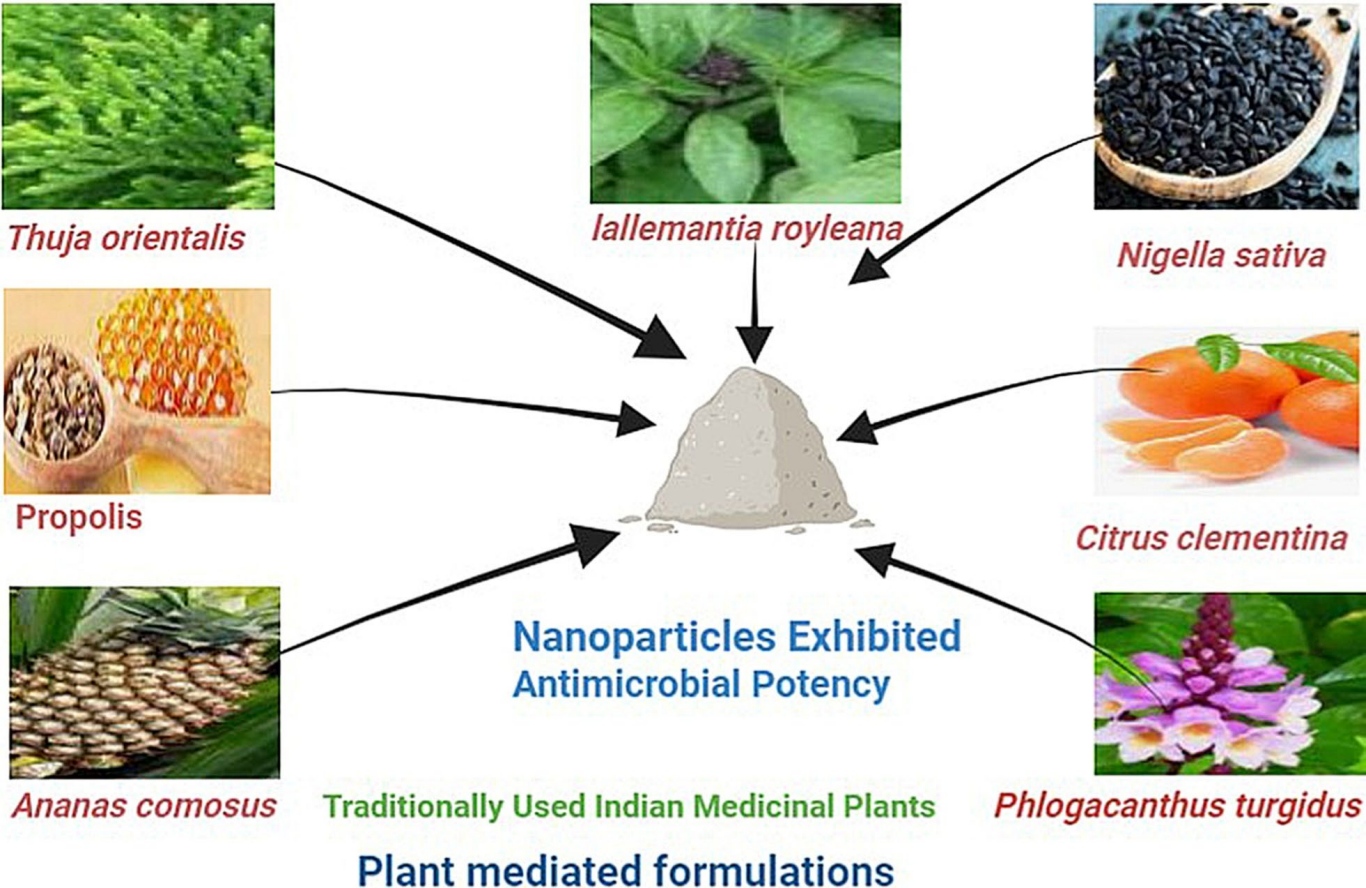
Nano formulations synthesized through plant-mediated methods demonstrate remarkable effectiveness in combating microbial infections

The egg extract of apple snail (*Pomacea canaliculata*) is harnessed for environmentally friendly silver nanoparticle (AgNPs) synthesis, containing proteins within a 24–65 kDa range that demonstrate reduction capabilities; these resulting AgNPs exhibit antibacterial efficacy against both Gram-positive *Staphylococcus aureus* and Gram-negative *Escherichia coli*, highlighting the potential for green synthesis of small-sized antibacterial AgNPs using the snail extract [103]. *Oscillatoria limnetica* cyanobacterial extract facilitates green synthesis of silver nanoparticles, serving as a reducer and stabilizer; the resulting quasi-spherical Ag-NPs (3.30 to 17.97 nm) possess robust antibacterial and cytotoxic effects against multidrug-resistant bacteria and cancer cells, while maintaining low toxicity to human red blood cells at lower concentrations, offering an eco-friendly alternative [104]. The eco-friendly synthesis of metallic nanoparticles through a blend of plant phytochemicals and microbial enzymes presents a sustainable and economical approach, where medicinal plants provide diverse phytochemicals, including alcohols, phenols, terpenes, alkaloids, saponins, and proteins, while microbial enzymes serve as efficient reducing and stabilizing agents throughout the nanoparticle formation process [105]. In the context of global agriculture's challenges from climate change, including abiotic and biotic stresses, utilizing nanomaterials from plant, animal, and fisheries waste for synthesizing metallic and non-metallic nanoparticles becomes crucial for enhancing stress resistance, pest management, nutrient efficiency, and resilience in climate-smart agriculture across crops, livestock, and fisheries [106]. The synergistic utilization of medicinal and aromatic plants (MAPs) along with nanotechnology, involving plant extracts and essential oils for nanoparticle synthesis, holds promise for healthcare advancements, enabling efficient phytomolecule delivery, novel nano-material synthesis, and exploration of MAPs' potential molecules (Fig. 6) [107].

Utilizing traditional Indian medicinal plants, inorganic nanoparticles are bio-fabricated through plant phytochemicals acting as reducing agents, with their non-toxic nature and synergistic interaction with precursor ions being critical for nanoparticle synthesis, offering diverse potential applications in healthcare and other sectors [108]. The interaction between metal nanoparticles and cellular components, including DNA and RNA, leading to cellular process modifications, is a well-explored area, with metal nanoparticles readily accessing bacterial cytoplasm and generating active oxygen species in the periplasmic space due to metal influence being significant factors [109].

### Metallic nanotechnology

Green and cost-effective biosynthesis of gold and silver nanoparticles employs reducing and stabilizing agents from diverse sources such as plants, microorganisms, and marine products, through one-pot or multistep processes at varying conditions, while chitosan, a natural cationic polysaccharide, gains prominence for its role in synthesizing biogenic metallic nanoparticles with potential nanomedicine applications as narrated in Table 1 [110]. Metal and metal oxide nanostructures are emerging as antibiotic alternatives, with their versatility and compatibility as nanocarriers being of keen scientific interest; these nanoparticles effectively inhibit bacterial growth, demonstrating substantial antibacterial, antifungal, antiviral, and antiparasitic potential against antibiotic-resistant microorganisms [111]. Enhancing silver diamine fluoride'seffectiveness involves employing topical cariostatic agents for managing dental caries in children; silver diamine fluoride proves practical for arresting caries and minimizing extensive treatment risks, while optimizing selenium nanoparticles' use alongside or instead of ionic silver, and exploring additional antimicrobial metal nanoparticles, considering optimal physicochemical parameters, presents potential strategies [112]. The utilization of polysaccharides as green reducing agents in metallic nanoparticle synthesis has garnered significant attention due to their versatile applications, exemplified by oxidized pullulan capped silver nanoparticles (OxP-AgNps) exhibiting potent antibacterial and antifungal activity against *Staphylococcus aureus, Escherichia coli, Pseudomonas aeruginosa,* and *Candida albicans*, while also demonstrating efficient photocatalytic degradation of water-soluble pollutants like methylene blue [113].

**Table 1:** Metallic nanotechnology and their applications

S.N	Nanotechnology	Applications	Authors
1	Silver and gold nanoparticles	Nanocomposites in nanomedicine	Katas et al. [110]
2	Metal and Metal oxide Nanostructures	Applied as alternatives of antibiotics	Kotrange et al. [111]
3	Amelioration strategies for silver diamine fluoride	Nanotechnology	Almuqrin et al. [112]
4	Silver nanoparticles	Covered by oxidized pullulan and used	Constantin et al. [113]
5	Silver and gold nanoparticles	Efficacy towards antibacterial	Soliman et al. [114]
6	Noble metallic nanoparticles	In agriculture	DAM et al. [115]
7	Photochemically prepared AgTiO2 membranes	Antibacterial properties of	Rahim et al. [116]
8	Silver nanoparticles	Antifungal activity	Sarjuna et al. [117]
9	Starch coated copper nanoparticles	Pancreatic cancer cells	Ilbasmis-Tamer et al. [118]
10	Metal nanoparticles	Pharmacological applications	Khandel et al. [119]
11	Nanoplatforms	Sepsis management	Luo [120]
12	Nanotechnologies	Food science	Nile et al. [121]
13	Antibiotic resistance	In aquaculture and aquatic organisms	Okeke et al. [122]
14	Immunomodulatory biomaterials	Implant-associated infections	Dong [123]
15	Phytochemical mediated synthesis of AgNP (silver nanoparticle)	Antimicrobial	Shaikh et al. [124]
16	Metal oxide nanoparticles	Wastewater disinfection	Gebre et al. [125]
17	Metal-based nanoparticles	Food packaging	Kumar et al. [126]
18	Marine derived biosurfactants	Microbially produced biosurfactants	Tripathi et al. [127]
19	Nanotechnology	Help to combat COVID-19	Campos et al. [128]
20	Synergism between metallic nanoparticles and antibiotics	Study synergism effects	Agreles et al. [3]
21	Nanoparticles	Environmental remediation potential	Rafeeq et al. [129]
22	Plasmonic Nanoparticle-Based Techniques	Treatment of COVID-19	Yakoubi et al. [130]
23	Silver nanoparticles by waste materials	Antimicrobial, antifungal, antiviral, and anticancer agents	Sharma et al. [4]
24	Functionalized silver nanoparticles	Eco-friendly fabrication of antibacterial cotton fibers	Xu et al. [6]
25	Metallic nanoparticles	Anti-plasmodial and mosquitocidal	Chandana Kulkarni et al. [131]
26	Celluloses as support materials	Antibacterial agents	Hasan Ahmad et al. [132]
27	Biosynthesis of nanoparticles and silver nanoparticles	Medical goods	Keat et al. [133]
28	Biologically synthesized silver nanoparticles	Antibacterial and Antibiofilm activities	Rolim et al. [134]
29	Nanoparticle and nanomaterial involvement during	Wound healing process	Pérez-Díaz et al. [135]
30	Nanocarriers for effective	Breast cancer therapy	Yap et al. [136]
31	Natural product-based nanomedicines for	Wound healing	Hajialyani et al. [137]
32	Nanomaterials as drug delivery systems with	Antibacterial properties	Khorsandi et al. [2]
33	Natural products with metal-binding properties as	Antibacterial agents	Dandawate et al. [138]
34	Noble metal nanoparticles	Medicine	Rai et al. [1]
35	Silver nanoparticles	Therapy of tuberculosis	Tăbăran et al. [139]

*Trichoderma saturnisporum* was utilized to create silver (Ag) and gold (Au) nanoparticles with enhanced productivity. The antibacterial efficacy of Ag-NPs and Au-NPs was evaluated against methicillin-sensitive and methicillin-resistant *Staphylococcus aureus, Pseudomonas aeruginosa,* and *Klebsiella pneumoniae*, demonstrating their potential as beneficial nanocompounds for medical applications through green synthesis [114]. Noble metallic nanoparticles exhibit antimicrobial effects against multidrug-resistant pathogens and confer plant protection, while nanobiosensors and nanodevices find application in agro-based domains like plant disease diagnosis and nanoparticle-mediated material delivery, showcasing the potential for noble metallic nanoparticles to revolutionize modern agricultural practices and drive sustainable precision agriculture trends [115]. Utilizing a photochemical process, AgTiO_2_ membranes were prepared by applying silver to ceramic supports coated with TiO_2_ powder through photoreduction with CH_3_COOAg solution at room temperature, resulting in AgOx and metallic silver detected on TiO_2_ via XPS analysis; direct contact with AgTiO_2_ exhibited *E. coli* eradication, suggesting potential for enhancing membrane antibacterial activity through AgTiO_2_ coating [116]. Two types of novel ternary deep eutectic solvents were developed. They act as a better solvent media for the synthesis of silver nanoparticles, and the synthesized nanoparticles show antifungal behaviors against some microbes [117], refer Table 1 for more details.

### Efficacy of metal based nanotechnology

A durable starch-coated copper nanoparticle (Cu-SNPs) formulation was studied for its apoptotic and necrotic impact on the Capan 1 pancreatic cancer cell line, demonstrating potent antimicrobial efficacy against *Staphylococcus* and *Enterococcus*, while in vitro cell culture assays revealed non-cytotoxic effects of Cu-SNPs across varying concentrations (6.25–100 ppm) on L929 fibroblasts and Capan 1 pancreatic cancer cells to varying degrees [118]. Metal nanoparticles including gold (Au), silver (Ag), lead (Pb), platinum (Pt), copper (Cu), iron (Fe), and metal oxides like titanium oxide (TiO) and zinc oxide (ZnO), synthesized through diverse methods including biological routes, present potential pharmacological applications in nanopharmaceutical products, explored alongside compound mechanisms and reduction processes, considering their current market status [119]. Nanoplatforms offer solutions to sepsis, a life-threatening condition involving uncontrolled immune responses to infection, by addressing clinical challenges like delayed diagnosis and immune disorder management; these well-designed nanoplatforms show promise in overcoming barriers and advancing therapeutic concepts for effective sepsis management [120]. In food science, nanotechnologies are utilized to enhance bioavailability, taste, texture, and consistency by altering particle size, cluster formation, and surface charge of food nanomaterials, alongside nanodelivery of nutraceuticals, synergistic food protection, and nanosensors in smart packaging to monitor stored food quality, while considering the assessment of nanomaterial impacts on biological systems [121]. The rapid growth of aquaculture for global food security necessitates exploring ecofriendly nanotechnological strategies such as nanodrug delivery, vaccine delivery, nanoformulations, and nanosensors to combat antibiotic resistance in aquaculture and aquatic organisms, while addressing potential public health risks associated with nanoparticles and implementing measures for sustainable management [122].

In the face of rising implant-associated infections (IAIs) and resilient bacteria on implant surfaces, creating effective anti-infective biomaterials requires considering both bacteria-killing capabilities and immune system modulation, recognizing the limitations of conventional approaches, highlighting the need for scientific guidance to advance innovative strategies in this field [123]. Over the past decade, worldwide biosynthesis of silver nanoparticles (AgNPs) through diverse plant species has been extensively studied, as revealed by bibliometric analysis, while the feasibility of plant-mediated nanoparticle synthesis and its applications has garnered interest, addressing engineering, economic, and environmental factors [124]. Metal oxide nanoparticles have traditionally been created through physical and chemical techniques, with distinct agents for reduction and stabilization. However, a sustainable approach has emerged, involving biological synthesis via plants, algae, and microbes, yielding nanoparticles that excel in functions like adsorption, membrane technology, photocatalysis, and wastewater disinfection [125]. Effectively storing excess food and its products while preserving freshness and nutrients could significantly enhance the food production industry. Stable and eco-friendly nanoparticles (NPs) have the potential to enhance various aspects of food packaging, from freshness indicators to mechanical properties and antibacterial characteristics [126].

Microbially produced biosurfactants sourced from renewable materials offer eco-friendly alternatives to petroleum-based surfactants, owing to their biodegradability. Particularly, marine-derived biosurfactants, often generated by non-pathogenic organisms, hold promise for commercial applications, with the potential for robust performance under extreme conditions like temperature, pH, and salinity [127]. Nanotechnology presents novel prospects in preventing, diagnosing, and treating viral infections like COVID-19. It involves developing nano-materials for disinfection, diagnostics, personal protection, treatments, and vaccines, while also necessitating the resolution of associated challenges and limitations [128]. Nanoscale manipulation through nanotechnology holds promise for addressing environmental challenges, with capping agents bolstering nanoparticle bioactivity by shielding them from the surroundings. Explored further are the applications of capped nanoparticles, including strategies to enhance their catalytic prowess, in tackling environmental pollution [129]. Plasmonic nanoparticles have been harnessed to create platforms for detecting, treating, and preventing SARS-CoV-2, showcasing their potential in innovative COVID-19 point-of-care diagnostics. The article emphasizes the significance of metallic plasmon NPs, explores existing hurdles, and looks ahead to clinical integration and commercialization of these diagnostic and therapeutic approaches to combat the COVID-19 pandemic [130].

Malaria, a mosquito-borne infectious disease, is prevalent in tropical and subtropical regions. Current research explores metallic nanoparticles for their potential in managing malaria through anti-plasmodial and mosquitocidal activities [131]. Examining performance stability, processing simplicity, cytotoxicity, and antibacterial efficiency, this study assesses the pros and cons of biocompatible antibacterial cellulose biocomposites. The study also suggests novel approaches to create longer-lasting and more effective biocomposites to fulfill increasing versatile application needs [132].

Measuring under 100 nm and composed of 20–15,000 silver atoms, silver nanoparticles capitalize on silver's compelling nanoscale attributes. Their increasing application in consumer and medical goods underscores their contemporary importance [133]. The simplicity and cost-effectiveness of biological methods have led to widespread investigation into metallic nanoparticle synthesis. Notably, AgNPsproduced by various biological sources like green tea, dill, and *S. hirsutum* exhibit significant antioxidant and antibacterial properties, holding promise in addressing antibiotic-resistant bacteria and biofilms [134]. Leveraging nanotechnology presents a powerful approach to advance wound healing by developing antibacterial nanomaterials to prevent infection and designing nanomaterials that promote cell growth, angiogenesis, and inflammation control for improved wound recovery; significant strides have been made in the last decade, utilizing nanoparticles to enhance wound healing through antimicrobial properties, increased cell proliferation, facilitated angiogenesis, controlled extracellular matrix formation, and anti-inflammatory modulation [135]. Despite notable progress in diagnosing and treating breast cancer, it continues to impact millions of women worldwide. Nanoformulations show enhanced effectiveness against preclinical breast cancer models, but additional clinical investigations are essential to validate their efficacy and safety for human use [136].

The complex sequence of biochemical and cellular events in wound healing aims to restore skin and subcutaneous tissue integrity. Plant extracts and their natural compounds, incorporated into nanoformulations, have shown significant potential in wound management, suggesting their potential as future pharmaceutical treatments [137]. Natural products with metal-binding attributes, notably polyphenols, quinones, 3-acyltetramic, and -tetronic acids, present potential as antibacterial agents, showing promise in drug development against diverse bacteria; synthesizing complex or unique natural products through synthetic techniques has been instrumental in addressing challenges in this area [138]. Employing silver nanoparticles introduces a groundbreaking strategy for addressing drug-resistant tuberculosis, showing potential to overcome limitations of existing organic antibiotics; while silver nanoparticles (AgNPs) offer promise for treating mycobacterial-related illnesses, their viability depends on resolving key therapeutic obstacles, including delivery constraints, variable intramacrophagic antimycobacterial efficacy, and residual toxicity [139].

## Regulatory framework and ethical consideration

Establishing protocols to clarify the physical–chemical properties of investigational pharmaceuticals particularly nanomedicines and nanocarriers in clinical trials is a necessary endeavour. In the midst of developing nanotechnology breakthroughs, regulatory criteria and rules need to be mapped to address crucial quality features and navigate obstacles, guaranteeing standardised procedures and prompt approvals [140]. Current hurdles to the translation of nanomedicines from bench to the clinic is the task. The need for clear regulatory guidelines to resolve obstacles in drug development and provides important factors for clinical translation of nanomedicine. It emphasises how crucial it is to pinpoint crucial quality characteristics, use suitable analytical techniques, guarantee batch consistency, and work closely with regulatory bodies in order to enable effective development [141]. Regulatory safety evaluation of nanomedical products has to be done effectively. Innovative medications can be made possible by nanotechnologies, but current safety assessment techniques are not up to par, especially when it comes to taking into account the special characteristics of nanomedicines. Important information is provided in this DDTR special issue on the REFINE initiative to improve safety evaluation techniques for nanomedical devices and close regulatory gaps [142].

### Green biomaterials: fundamental principles

Through the integration of knowledge and technology for need-based change that benefits society and the environment, Green Biomaterials seeks to revolutionise the area. The manuscript emphasises the synthesis and development of materials in line with green and healthy principles, while also defining and exploring the field of green biomaterials. It gives scientists and researchers a venue to share their most recent findings in the field, encouraging interdisciplinary cooperation and the advancement of ecologically friendly chemical industry technology as noted in Table 2 [143].

**Table 2:** Fundamental and application-oriented principle of green biomaterials

Fundamental principles	Application-oriented principles
Prevention of toxic waste	Designing non-toxic products
Waste-free reactions	Employing real-time and in situ analysis to prevent toxic reactions/materials
Utilization of safer and greener solvents	
Adoption of renewable and non-toxic feedstocks	Creating biodegradable and biocompatible products/materials
Application of nature-inspired protocols	Designing multifunctional non-toxic bioactive materials
Use of natural and sustainable components	Developing bioactive materials with easy and rapid clearance from physiological systems,
Reduced carbon footprint	Designing (bio)-recyclable products/materials
Life cycle sustainability	Conducting rapid life cycle assessments for overall environmental impact, and
Resource conservation	
Energy-efficient production	

### Environmental impact of the synthesis methods and the use

Navigating the formidable challenge of cellular internalization seems akin to tackling a mission impossible. How rigid porphyrin structures (H2TMP) and metal–organic frameworks (MOFs) can hybridise to speed up drug loading and release into cells. Cell lines were used to synthesise and characterise a variety of MOFs, showing enhanced drug loading capacity and increased relative cell viability for H2TMP-coated nanocarriers [144]. Metallic nanostructure-based aptasensors offer a robust platform for the detection of proteins. Aptasensor development has been driven by the need for quick, affordable, and extremely sensitive protein target detection in food, environmental monitoring, and healthcare. Through signal amplification and transduction mechanisms, integrating high-affinity aptamers with different metal-based nanomaterials, such as noble metal nanoparticles, metal oxides, and graphene-conjugated structures, improves sensitivity [145]. Hepatobiliary illness is a serious health risk that is impacted by things like longer life expectancies and sedentary habits. Research on the pharmacological actions, components, and processes of *CurcumaeRhizoma* (EZ), a staple of traditional Chinese medicine (TCM), has demonstrated its effectiveness in treating hepatobiliary illnesses. These mechanisms involve antioxidant and anti-inflammatory pathways. While encouraging, more research is required to alleviate hepatotoxicity and expand therapeutic applications [146]. Parkinson's disease (PD) and other neurodegenerative disorders are largely influenced by inflammation and neuroinflammation. Promising anti-inflammatory and ROS-scavenging nanomaterials may help reduce Parkinson's disease. This review delves into the pathophysiology of Parkinson's disease (PD), focused delivery approaches, and the latest advancements in nanomaterials. It classifies these materials according to their methods of PD relief, which include anti-inflammatory and interference with α-synuclein pathology [147].

Since the immunosuppressive bone microenvironment limits the effectiveness of current treatment, bone metastasis is a leading cause of death in prostate cancer (PCa). In order to improve immunotherapy for patients with bone metastatic PCa, this study created a bone-targeted nano-delivery system that specifically targeted PCa cells, caused immunogenic cell death, and functioned as a nano-regulator by obstructing the immunosuppressive TGF-β signalling pathway [148]. Research is influenced by both hemostatic and anticoagulant characteristics, which present a dual challenge for blood-contacting materials. Because thrombus formation on biomedical equipment might affect their performance, coatings that are super-lubricated or super-hydrophobic as well as those that administer antithrombotic medications have been developed. Furthermore, sticky hydrogels, biological compounds, and hemostatic biomaterials with porous architectures are investigated for quick hemostasis. This paper provides a thorough overview of surface-functionalized designs for biomedical engineering, covering topics such as preparation techniques for anticoagulant coatings and the state of hemostatic materials today [149]. The green manufacturing of metal oxide nanoparticles by nanotechnology holds great potential for a variety of scientific uses. This work demonstrated increased antibacterial and anticancer activity of green-synthesised Bismuth oxide nanoparticles, including silver (Ag) and copper (Cu) doping. The results point to the promise of these nanoparticles in biomedicine while highlighting the necessity of more in vivo research and mechanistic knowledge for potential uses in the future [150].

## Discussion

The analysis emphasises how urgently creative approaches to the fight against antibiotic resistance are needed. Promising antibacterial characteristics of bioengineered metallic nanoparticles derived from nature highlight an emerging field of study. Their various synthesis techniques and modes of action present opportunities for scalable solutions and personalised therapy. To fully utilise them, however, issues like toxicity and regulation must be carefully considered.

**Fig. 7:**
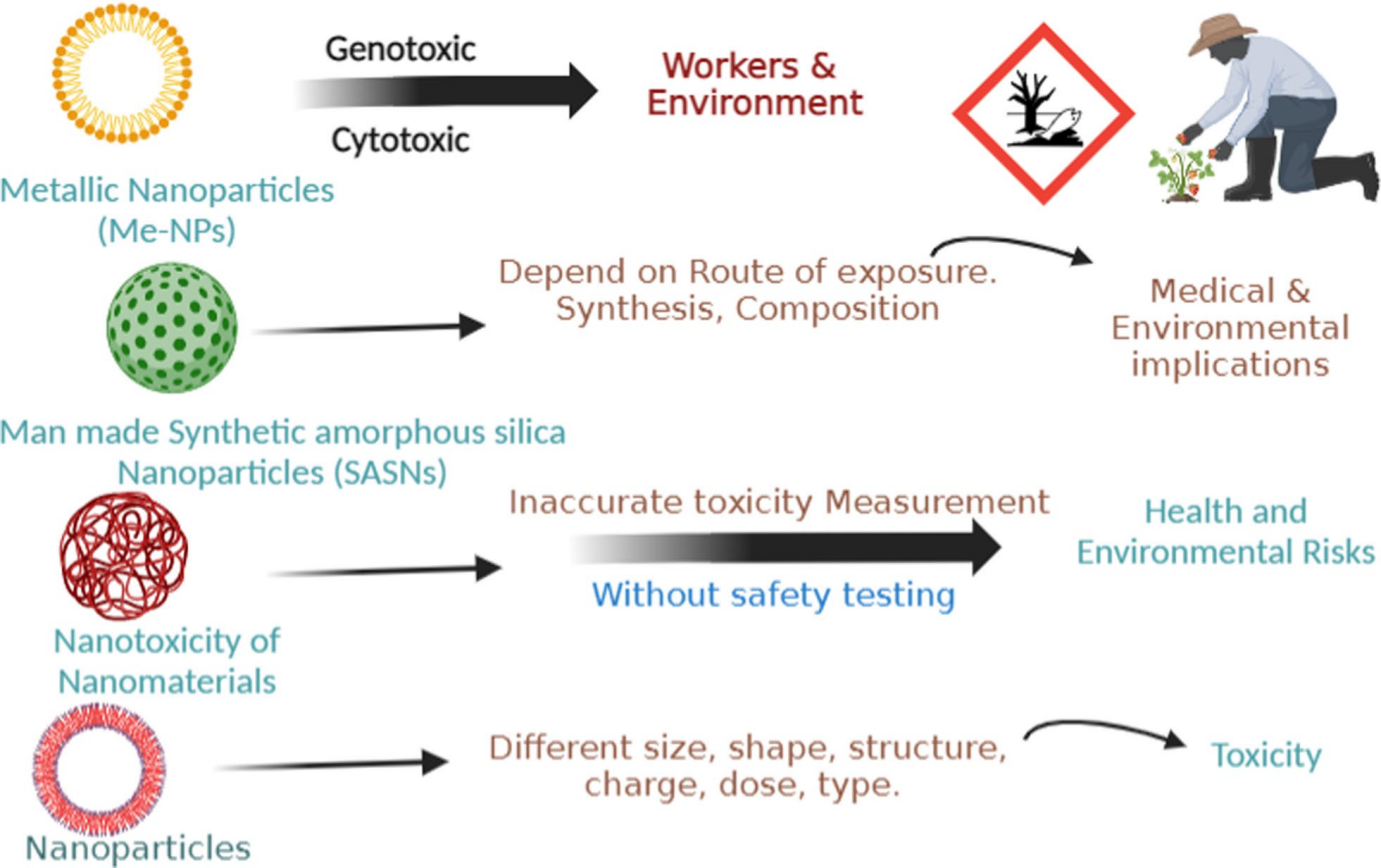
Adverse effects of bioengineered metallic nanoparticles

The toxicity of intentionally created metallic nanoparticles (Me-NPs), such as silver, gold, iron oxide, copper oxide, nickel oxide, manganese oxide, lead oxide, and zinc oxide, was methodically investigated (Fig. 7). The study also showed that well-composed mixtures of bioactive substances could be given in safe amounts to reduce the toxicity of Me-NPs [151]. Biomedical and environmental implications of synthetic amorphous Silica nanoparticles also observed. The copious natural silica and silicates found in the Earth's crust are not the same as synthetic amorphous silica nanoparticles (SASNs), which are produced for industrial and pharmaceutical uses. The study emphasises the requirement for standardised characterization in reporting biological and environmental behaviours and emphasises the need of comprehending SASN composition, synthesis, and environmental exposure for correct toxicity evaluation [152]. The field of nanotoxicology has seen significant investigation over the last 20 years, which has led to a greater understanding of the mechanisms and evaluations of nanotoxicity. Because of their special qualities, nanomaterials (NMs) have a lot of potential applications; yet, because of their small size and exposure, they can pose health and environmental risks. The fact that many NMs are utilised in industrial and medical applications without first undergoing safety testing emphasises the necessity of accurate toxicity measurements and the standardised processes described in this thorough analysis [153]. Structural parameters of nanoparticles affecting their Toxicity, because nanoparticles (NPs) are more poisonous and have higher chemical activity than bulk materials, there is growing interest in using NPs in biomedical applications. This has led to worries about safety. This study delves into the mechanisms and factors that impact nanoparticle (NP) toxicity, highlighting the importance of comprehending these aspects to create safer NPs for uses in gene transfer, medication delivery, imaging, and anti-SARS-CoV-2 vaccine development. The article explores the toxicity of several types of nanoparticles (NPs) taking into account physiochemical characteristics such as surface charge, size, and shape [154].

Drug resistance and low solubility are two issues that traditional cancer nanomedicine therapies encounter, and the disease nevertheless poses a threat to world health. With its increased efficacy and specificity, nanomedicine is a game-changer in the treatment of cancer. It addresses a number of modalities and highlights the importance of resolving toxicity issues and improving drug delivery techniques [155]. With a focus on cost-effectiveness and biocompatibility, this perspective piece investigates the possibilities of surface engineering with natural and eco-friendly materials for CRISPR delivery. The state of the field, obstacles to be overcome, and potential applications of natural components in addressing conventional delivery constraints [156]. The difficulties in improving treatment efficacy while discussing the usefulness of anticancer nanomedicines in reducing the negative effects of chemotherapy drugs. It offers insights into design concepts and tactics for better cancer treatment. It focuses on stimuli-responsive nanomedicines intended to address anomalies in the tumour microenvironment [157].

## Conclusion

In summary, this review highlights the promising landscape of metallic nanoparticles (MNPs) synthesized from natural products, emphasizing their current status and future prospects. MNPs, sourced from diverse natural origins, demonstrate significant potential as versatile agents with antimicrobial properties against various pathogens. The inherent bioactive compounds in natural products facilitate MNP synthesis and stabilization, while their unique physicochemical attributes contribute to effective antimicrobial mechanisms.

The fusion of nanotechnology with nature-derived MNPs presents a hopeful solution to the challenges posed by antimicrobial resistance. However, this emerging field requires ongoing research efforts to standardize synthesis methodologies, thoroughly evaluate toxicity profiles, and establish optimal application protocols. Moving forward, the incorporation of MNPs into clinical and industrial settings necessitates further exploration of pharmacokinetics, safety considerations, and potential synergies with existing treatments.

In conclusion, MNPs derived from natural products offer a compelling pathway to revolutionize antimicrobial strategies, providing a sustainable, efficient, and innovative approach to combating infectious diseases and safeguarding public health. Future research should focus on addressing these identified challenges and implementing strategies to maximize the efficacy and safety of MNPs in diverse applications.

## Data Availability

Not applicable.
